# Uterine Polypus—A Case

**Published:** 1886-01

**Authors:** L. G. Hardman

**Affiliations:** Harmony Grove, Ga., Chairman Section on Gynæcology for Ninth Congressional District


### UTERINE POLYPUS—A CASE.

BY L. G. HARDMAN, M. D., HARMONY GROVE, GA.,

CHAIRMAN SECTION ON GYNÆCOLOGY FOR NINTH CONGRESSIONAL DISTRICT.

Mrs. G———, age 49 years; married; mother of several healthy children; previous health and history good. The present trouble dates back something over two years ago. She was taken with violent and alarming hemorrhage of the uterus while in the field at work. It continued more or less for three weeks. Supposing the hemorrhage to have been caused by the "change of life," the family physician prescribed tonics, and with only partial relief, and that for a short time. Another physician was consulted, who examined the patient and pronounced it a hopeless case of uterine cancer, and gave the case up after some heroic attempts to "burn it off." With exception of the above treatment, as detailed to me by the woman herself, the history of the case is that of continued hemorrhage. Sometimes the bleeding would stop for a few days or a week, to return again. The patient was confined to the bed all the time from weakness resulting from the loss of blood. This, together with the unfavorable prognosis of her former medical attendant, rendered her very despondent, nervous, and, at times, hysterical. At my first visit she presented a miserable appearance, indeed. On examining the case, I found a polypus which occupied the upper portion of the vagina and was attached to the inner uterine surface about the junction of the cervix with the body by a short, thick pedicle apparently about a half inch in diameter. I informed the husband of the nature of the case, and told him the only thing that could be done was to remove it by an operation, to which ready assent was given. On the fifth of May, one month after the first examination, I made the operation with an ecrasure. There was but little pain, and no hemorrhage following the removal of the tumor.

The vagina was now tamponed with cloth saturated with solution of tanic acid, and the patient was given fifteen drops tinct. iron and fifteen drops tinct. cannabis indica every three hours, and left under the care of her physician, Dr. Lockhart.

The case made a good recovery, and was dismissed well six weeks after the operation.

